# Alterations in ROS Activity and Lysosomal pH Account for Distinct Patterns of Macroautophagy in LINCL and JNCL Fibroblasts

**DOI:** 10.1371/journal.pone.0055526

**Published:** 2013-02-07

**Authors:** José Manuel Vidal-Donet, Jaime Cárcel-Trullols, Bonaventura Casanova, Carmen Aguado, Erwin Knecht

**Affiliations:** 1 Laboratory of Cellular Biology, Centro de Investigación Príncipe Felipe, Valencia, Spain; 2 Centro de Investigación Biomédica en Red de Enfermedades Raras (CIBERER), Valencia, Spain; 3 Neurology Department, Hospital La Fe, Valencia, Spain; University of Melbourne, Australia

## Abstract

Neuronal Ceroid Lipofuscinoses (NCL) are lysosomal storage disorders characterized by the accumulation of lipofuscin within lysosomes. Late infantile (LINCL) and juvenile (JNCL) are their most common forms and are caused by loss-of-function mutations in tripeptidyl peptidase 1 (TPP1), a lysosomal endopeptidase, and CLN3 protein (CLN3p), whose location and function is still controversial. LINCL patients suffer more severely from NCL consequences than JNCL patients, in spite of having in common an abnormal accumulation of material with a similar composition in the lysosomes. To identify distinctive characteristics that could explain the differences in the severity of LINCL and JNCL pathologies, we compared the protein degradation mechanisms in patientś fibroblasts. Pulse-chase experiments show a significant decrease in protein degradation by macroautophagy in fibroblasts bearing TPP1 (CLN2) and CLN3p (CLN3) mutations. In CLN2 fibroblasts, LC3-II levels and other procedures indicate an impaired formation of autophagosomes, which confirms the pulse-chase experiments. This defect is linked to an accumulation of Reactive Oxygen Species (ROS), an upregulation of the Akt-mTOR signalling pathway and increased activities of the p38α and ERK1/2 MAPKs. In CLN3 fibroblasts, LC3-II analysis indicates impairment in autophagosome maturation and there is also a defect in fluid phase endocytosis, two alterations that can be related to an observed increase of 0.5 units in lysosomal pH. CLN3 fibroblasts also accumulate ROS but to a lower extent than CLN2. TPP1 activity is completely abrogated in CLN2 and partially diminished in CLN3 fibroblasts. TPP1 cleaves small hydrophobic proteins like subunit c of mitochondrial ATP synthase and the lack or a lower activity of this enzyme can contribute to lipofuscin accumulation. These alterations in TPP1 activity lead to an increased ROS production, especially in CLN2 in which it is aggravated by a decrease in catalase activity. This could explain the earlier appearance of the symptoms in the LINCL form.

## Introduction

Neuronal Ceroid Lipofuscinoses (NCL) make up the most common group of inherited neurodegenerative disorders of childhood. Many mutations in at least eight different genes are responsible for causing NCL. Among those, *CLN2/TPP1* and *CLN3* mutations give rise to the most current types of NCL disorders, the Late Infantile (LINCL; OMIM 204500) and the Juvenile (JNCL, OMIM 204200) forms, respectively. Both are recessively inherited. childhood-onset neurodegenerative disorders characterized by progressive blindness, seizures, motor and cognitive decline and early death [1].

Tripeptidyl peptidase 1 (TPP1), the defective enzyme in LINCL, is a lysosomal aminopeptidase that removes tripeptides from the unmodified N-terminus of small proteins [2],[3]. The *CLN3*-encoded protein (battenin, also called CLN3p), which is defective in JNCL, is a highly conserved, ubiquitously expressed, multi-pass membrane protein that localizes in lysosomes and in other vesicular compartments [4]–[6], and its precise function is still controversial. Studies with mutated Btn1p, the ortholog protein of CLN3p in yeast, as well as subsequent analyses in patientś fibroblasts and human embryonic kidney cells with mutated CLN3p have shown disturbances in the maintenance of the vacuolar/lysosomal pH [7]–[9]. However, while most of the studies have described an increased pH, others reported a decrease and even others, and in spite of these studies, have considered that CLN3p plays no role in lysosomal pH homeostasis. In addition, other functions have been proposed for this protein, such as lysosomal amino acid transport, traffic of sphingolipids from the Golgi complex to the plasma membrane, regulation of cell membrane trafficking and endocytosis [10]–[15]. The presence of TPP1 and CLN3p, as well as of other CLN proteins such as CLN1 and CLN5, within the endosomal–lysosomal system, their traffic through the endoplasmic reticulum and Golgi system and, most importantly, the common phenotype observed in the different forms of NCL, suggest that they could participate at different stages in a common biological process that becomes altered [16]. In this sense, macroautophagy is a good candidate, since most CLN proteins are lysosomal and, under certain conditions, it can be considered a pro-survival process, even when it can also cause cell death [17].

Macroautophagy has been implicated in different neurodegenerative diseases such as Parkinson [18], [19], Huntington [20]–[22], Alzheimer [23], [24] and also NCL [25], [26], [27]. It is mostly, but not exclusively, a non-selective process that generates metabolites for cell survival and that is especially active at times of stress or nutritional deprivation. This pathway starts with the formation in the cytosol of an isolation structure of still unknown origin that engulfs cytoplasmic constituents, including organelles, which closes to form a double membrane autophagosome. By fusing with late endosomes and lysosomes, this autophagic vacuole matures to a single membrane autolysosome, acquiring in this way hydrolytic enzymes. Macroautophagy converges with the endocytic pathway, responsible for the internalization of extracellular and plasma membrane molecules, at the lysosome [28], where all this material is degraded by hydrolases with an optimal acidic pH [29]. Finally, the resulting metabolites are recycled back to the cell [17]. Macroautophagy is regulated under various stimuli by different signalling pathways, with mTOR occupying a central position in most of them [30]. One of these stimuli is the generation of Reactive Oxygen Species (ROS), which regulate macroautophagy usually through ATG4, a specific protease that controls autophagosome formation and maturation [31].

Here, using TPP1- (CLN2) and CLN3p- (CLN3) deficient fibroblasts, we have compared the alterations in protein degradation and the respective triggering events that could lead to a common phenotype. An important objective of our studies was to provide insights into the differences in the severity of both NCL forms. Our results support that an upregulated Akt-mTOR signalling pathway or an increased pH, in LINCL and in JNCL or in only JNCL, respectively, produces a common disruption in the normal functioning of macroautophagy and endocytosis that could account for the similarities in the phenotypes of the respective pathologies. In spite of macroautophagy/endocytosis being altered in both types of fibroblasts, there are some additional differences that could explain the earlier appearance of the symptoms in the LINCL form. Thus, total loss of TPP1 activity and accumulation of higher amounts of ROS in LINCL fibroblasts could both increase the accumulation rate of peroxidized lipids and lipofuscin in LINCL.

## Results

LINCL and JNCL are the most common forms of Neuronal Ceroid Lipofuscinoses. The accumulation within lysosomes of non-degraded molecules, mainly subunit c of mitochondrial ATP synthase, is a common trait in these diseases. In spite of this, LINCL and JNCL patients have different onset ages and lifespans. Therefore, we analyzed in CLN2 and CLN3 fibroblasts possible alterations in lysosomal cell processes involved in intracellular protein degradation that could account for the observed differences in LINCL and JNCL patients, respectively. We started with macroautophagy, because it is the main lysosomal protein degradation mechanism.

### Macroautophagy is Impaired in CLN2 and CLN3 Fibroblasts

Macroautophagy operates at low levels under basal conditions, but becomes strongly activated under starvation conditions. Thus, to analyze their possible alterations, we investigated both conditions (see Materials and Methods and below) to determine if they are detected under a low macroautophagic activity or only when a strong activation of macroautophagy is required.

**Figure 1:**
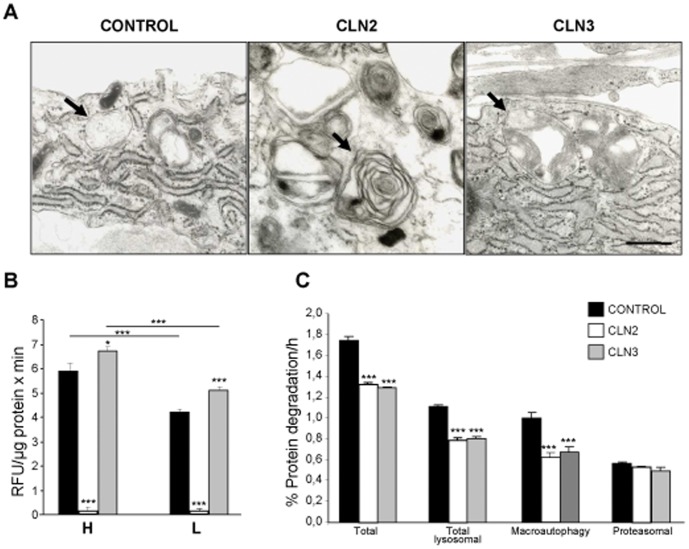
Lysosomal alterations in NCL fibroblasts. (A) Representative electron micrographs of control, CLN2 and CLN3 fibroblasts incubated in high proteolysis medium to activate autophagy. Arrows indicate lysosomal vacuoles. Bar: 0.5 µm. (B) TPP1 activity measurements. Control (black bars), CLN2 (white bars) and CLN3 (gray bars) fibroblasts were incubated under high (H) and low (L) proteolysis conditions as indicated. TPP1 activity and total protein were measured as described in Materials and Methods. Results are expressed as RFU values per minute and µg protein, and are the mean and S.D. from three separate experiments with triplicated samples. (C) Pulse-chase experiments. Labelling of human fibroblasts with [^3^H] valine in high proteolysis medium, measurements of degradation of long-lived proteins and the contribution of lysosomal (total and macroautophagy) and proteasomal pathways were carried out as described in Materials and Methods. Results are presented as the percentage of the labelled protein that is degraded per hour, and are the mean and S.D. from eight to fifteen separate experiments with duplicated samples. In B and C, stars immediately on top of bars indicate statistically significant differences from control values (*p<0.05 and ***p<0.005).

As expected [12], when compared with controls, both CLN2 and CLN3 fibroblasts accumulate autophagic vacuoles containing rests of organelles and membranes (arrows in Figure 1A). In addition, TPP1 activity, which is known to be completely abrogated in LINCL patients [2], could not be detected, as expected, in extracts from CLN2 fibroblasts previously incubated under high (H, KH) and low (L, KH supplemented with insulin and amino acids) proteolysis conditions and, thus, of high and low macroautophagic activity. In extracts from CLN3 fibroblasts, the *in vitro* activity of the enzyme at its optimal pH was significantly increased (about 15%) under both conditions in comparison to controls (Figure1B), suggesting an attempt to compensate for the defect in CLN3p. In addition, TPP1 activity increased under high proteolysis conditions in both control and CLN3 cell extracts, probably due to an adaptive response to conditions requiring a much higher proteolytic activity of lysosomes.

Next, we examined if the degradation of different pools of intracellular proteins was altered in CLN2 and CLN3 fibroblasts, analyzing the degradation of long-lived proteins under high proteolysis conditions in pulse-chase experiments. In addition to total protein degradation, we analyzed also separately the lysosomal (with 20 mM NH_4_Cl plus 0.1 mM leupeptin) and proteasomal (with 10 µM lactacystin) degradations as described previously [32]. Total degradation of long-lived proteins is diminished in CLN2 and CLN3 fibroblasts (about 20–25% inhibition, Fig. 1C). Total lysosomal degradation (macroautophagy and other lysosomal proteolytic pathways), which under these high proteolysis conditions mainly corresponds to macroautophagy, seems to account for the differences observed, since proteasomal degradation is not significantly affected. This conclusion is further supported by measuring the 3-methyladenine-sensitive protein degradation, which is considered to correspond to the contribution of macroautophagy to intracellular protein degradation [32]: there is a clear decrease (about 25%) in the amounts of long-lived proteins degraded by macroautophagy (*i.e.* 3-methyladenine-sensitive) in both CLN2 and CLN3 fibroblasts. These effects were almost non-existent when the degradation of long-lived proteins was analyzed in low proteolysis medium and when analyzing the degradation of short-lived proteins under both conditions (Figure S1). Since in all these cases the degradation of proteins in human fibroblasts occurs by pathways different from macroautophagy [32], [33] and we did not observe any significant alteration in this degradation in both CLN2 and CLN3 fibroblasts, it appears that macroautophagy is the main protein degradation pathway that is impaired in these cells, in agreement with the 3-methyladenine results (see above).

### Synthesis and Maturation of Autophagosomes are Decreased, Respectively, in CLN2 and CLN3 Fibroblasts

**Figure 2:**
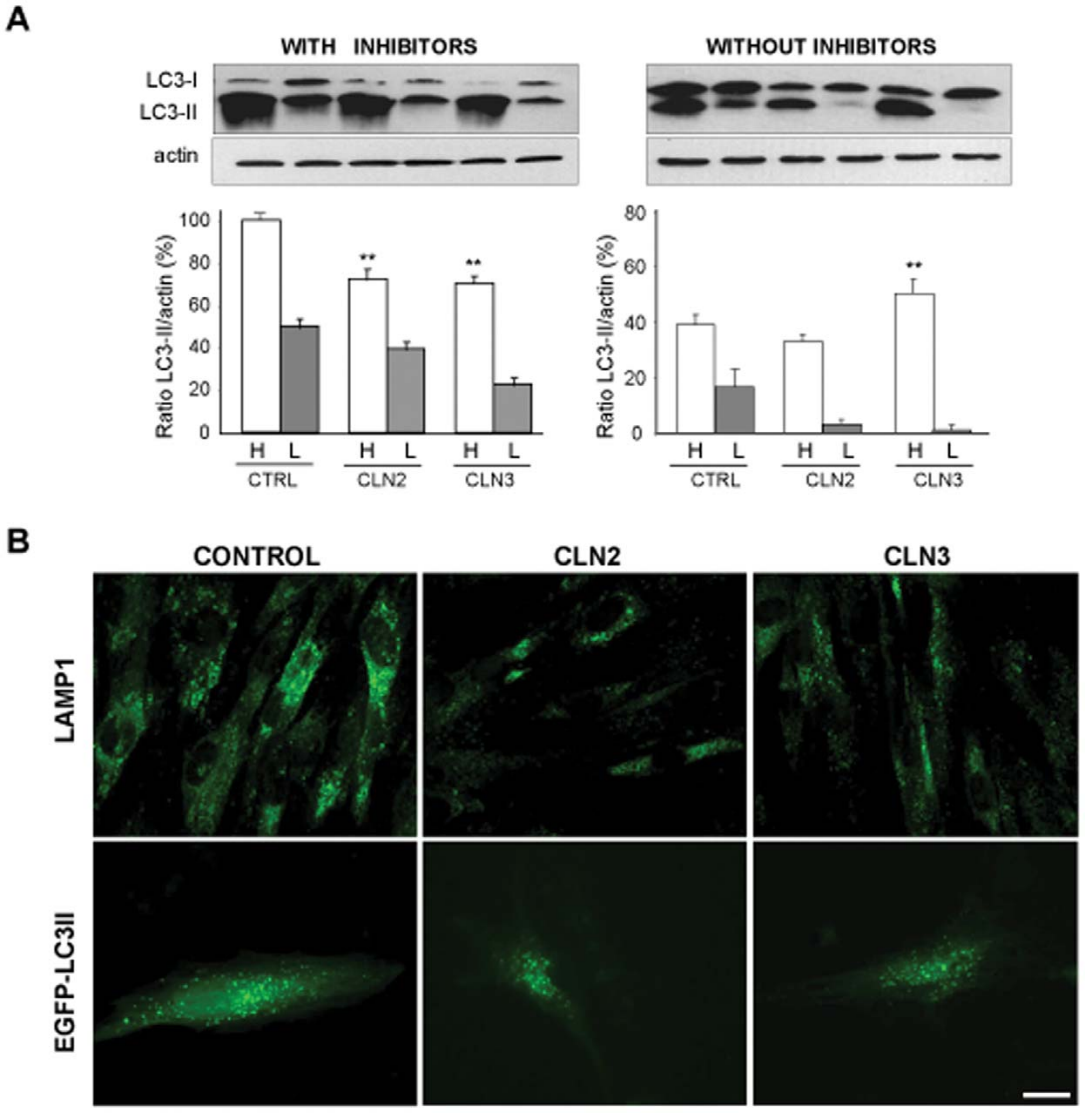
Reduced autophagosome formation in CLN2 and CLN3 fibroblasts and decreased autophagosome maturation in CLN3 cells. (A) Total extracts from control (CTRL), CLN2 and CLN3 fibroblasts incubated under high (H) and low (L) proteolysis conditions and with or without lysosomal inhibitors (see Material and Methods) were analyzed by western-blot using LC3 and, as a loading control, actin antibodies. The position of LC3-I, and LC3-II bands are indicated on the left. Densitometric measurements from three different experiments are shown below. Results are presented as LC3-II/actin ratios and are expressed as percentages of the control values with inhibitors under high proteolysis conditions. Stars indicate statistically significant differences under high proteolysis conditions from the corresponding control values (**p<0.01). (B) Representative fluorescence images of control, CLN2 and CLN3 fibroblasts incubated in high proteolysis medium for 2 h at 37°C. Upper micrographs: immunocytochemistry with anti-LAMP1. Lower micrographs: control, CLN2 and CLN3 fibroblasts, 48 h after transfection with pEGFP-LC3. Bar: 20 µm.

To analyze in more detail the step in the autophagic flux where macroautophagy is impaired, endogenous LC3-II was examined by immunoblotting with or without inhibitors of lysosomal proteases (Figure 2A). LC3-I is a microtubule-associated protein that is lipidated to LC3-II upon activation of macroautophagy. LC3-II is considered, under starvation conditions, a marker of macroautophagy, since it associates to preautophagosomal and autophagosomal membranes [34]. Using an LC3 antibody that recognizes both forms of LC3 (see Materials and Methods), we determined by densitometry the ratio of LC3-II to actin levels in cell extracts from control, CLN2 and CLN3 fibroblasts. In the presence of lysosomal inhibitors, and under conditions of high proteolysis (H, starvation), results show lower LC3-II protein levels both in CLN2 and CLN3 fibroblasts as compared to their controls (Figure 2A, left). These results indicate impairment in autophagosome formation in both NCL fibroblasts. Without protease inhibitors, LC3-II levels increased in CLN3 fibroblasts in high proteolysis medium, indicating that in these cells there is also a defect in autophagosome maturation under starvation conditions (Figure 2A, right). In all these experiments, the levels of the LC3-I bands in CLN2 and CLN3 fibroblasts were slightly, but consistently, reduced compared to control fibroblasts (see the upper band in the western blots). Since LC3-I is processed into LC3-II, this reduction could contribute to the observed decreased of macroautophagy in NCL fibroblasts. However, the significance of the differences in LC3-I levels among different cell types is not fully clear, because LC3-I is less stable, less immunoreactive and more sensitive to freezing-thawing and to degradation in SDS-sample buffer than LC3-II, and the appearance of the bands also depends on the primary antibodies used (and even on the antibody lot) and on the cell type and treatment.

The observed impairment in autophagic flux that occurs under starvation was also confirmed using the lysosomal marker LAMP1. The LAMP1-associated fluorescence was clearly decreased in CLN2 and CLN3 fibroblasts (Figure 2B, upper micrographs). Furthermore, under these conditions, a reduction in the number of autophagic vacuoles was evident in both CLN2 and CLN3 fibroblasts that transiently expressed EGFP-LC3 (Figure 2B, lower micrographs), since the number of fluorescent puncta decreased in CLN2 (70±15) and CLN3 (77±18), compared to control (152±20) fibroblasts. These results are also in agreement with an observed decrease in the fluorescence intensity of monodansylcadaverine, which stains autophagic vacuoles and lysosomes, in both CLN2 and CLN3 fibroblasts (Figure S2A and S2B).

### Macropinocytosis is Impaired in CLN2 and CLN3 Fibroblasts

**Figure 3:**
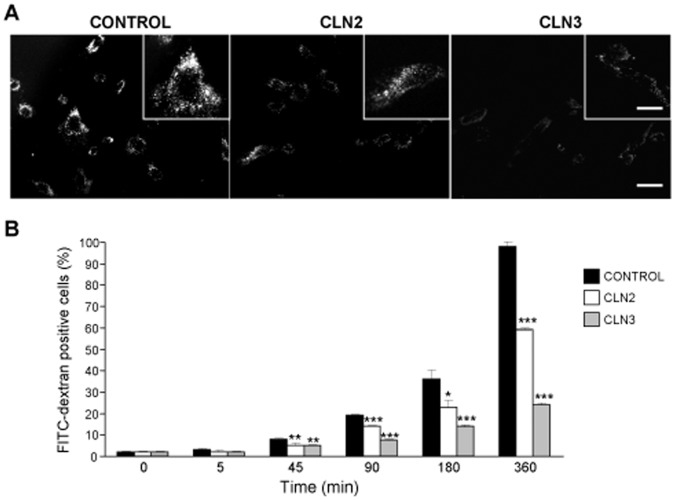
Macropinocytosis is reduced in CLN2 and especially in CLN3 fibroblasts. (A) Representative fluorescence microscopic images of control, CLN2 and CLN3 fibroblasts incubated at 37°C for 2 h with 0.5 mg/ml FITC-dextran, followed by a 30 min chase. Bar: 50 µm. Insets show cells at higher magnification. Bar: 150 µm. (B) To quantify endocytic internalization, control, CLN2 and CLN3 fibroblasts were incubated at 37°C for the indicated periods of time with 0.5 mg/ml FITC-dextran, washed and analyzed by flow citometry as described in Materials and Methods. Results indicate the percentage of fluorescently labelled cells, and are the mean and S.D. from three separate experiments with triplicated samples. Representative dot blots are shown in Figure S3A. Stars indicate statistically significant differences from the corresponding control values at each time (*p<0.05, **p<0.01 and ***p<0.005).

The autophagic and endocytic pathways converge into lysosomes to degrade their contents [28]. Since an impaired macroautophagy could affect the endocytic pathway, we tested macropinocytosis of FITC-dextran in NCL fibroblasts. Compared to controls, FITC fluorescence was lower in CLN2 and, especially, in CLN3 fibroblasts (Figure 3A). These results were confirmed by flow cytometric analysis at different times of internalization (Figure 3B and Figure S3A). Thus, for example, after 6 h of FITC-dextran internalization, the fluorescence in CLN2 and CLN3 fibroblasts was 66.3 and 28.3%, respectively, of control fibroblasts.

### The Akt-mTOR Pathway is Upregulated in CLN2 Fibroblasts

**Figure 4:**
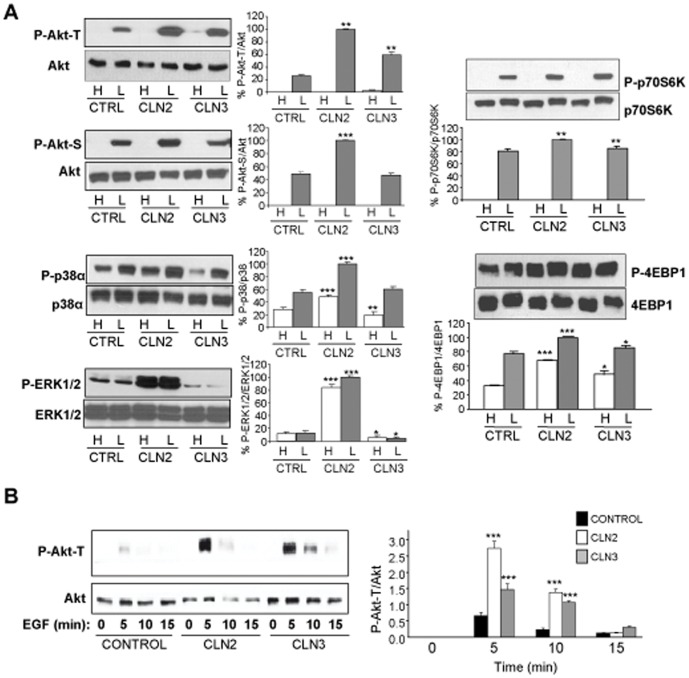
The Akt-mTOR pathway is more activated in CLN3 and especially in CLN2 fibroblasts. (A) Control (CTRL), CLN2 and CLN3 fibroblasts were incubated in high (H) and low (L) proteolysis media. Lysates were analyzed by western blot using antibodies against the phosphorylated forms of various proteins upstream and downstream of mTOR: Akt-Thr 308 (P-Akt-T), Akt-Ser 473 (P-Akt-S), p-38α (P-p38α), ERK 1/2 (P-ERK 1/2), p70S6K (P-p70S6K) and 4EBP1 (P-4EBP1) and their respective total proteins. The bands corresponding to the phosphorylated proteins, obtained from three different experiments, were densitometred and normalized to the corresponding bands of total protein. Data are plotted in percentage relative to the highest value. Stars indicate statistically significant differences from control values under high and low proteolysis conditions (*p<0.05, **p<0.01 and ***p<0.005). (B) Control, CLN2 and CLN3 fibroblasts were incubated for the indicated times in high proteolysis medium with EGF (100 ng/ml). Total extracts were analyzed using P-Akt-T and total Akt antibodies. A representative blot is shown. The bands corresponding to phosphorylated and total Akt were quantified by densitometry as in A). Stars indicate statistically significant differences from control values at each time (***p<0.005).

The Akt-mTOR pathway negatively regulates macroautophagy by inhibiting the synthesis of autophagosomes [30]. To examine its possible involvement in the observed decrease of macroautophagy in NCL fibroblasts, we analyzed the phosphorylation of Akt, an upstream activator of mTOR. We used two different stimuli to activate this pathway. As shown in Figure 4, after incubation with insulin and amino acids (A) or epidermal growth factor (B), Akt is more phosphorylated in CLN3 (at T308, but not at S463) but especially in CLN2 (both at T308 and S463) fibroblasts compared to controls. The two MAPKs p38α and ERK 1/2, which can also activate mTOR, are clearly upregulated in CLN2 fibroblasts, while the opposite occurs in CLN3 fibroblasts, except for p38α under low proteolysis conditions, where there was no difference with control fibroblasts (Figure 4A). The immediate substrates of mTOR, p70 S6 kinase (p70S6K) and 4E-binding protein 1 (4EBP1), were especially more phosphorylated in CLN2 fibroblasts compared to control fibroblasts (Figure 4A), although both proteins were also more phosphorylated, but to a slightly lower extent, in CLN3 fibroblasts, in agreement with the other data indicating an alteration of macroautophagy in all these cells.

### ROS Accumulates, Catalase Activity is Decreased and Cell Cycle is Altered in CLN2 Fibroblasts

**Figure 5:**
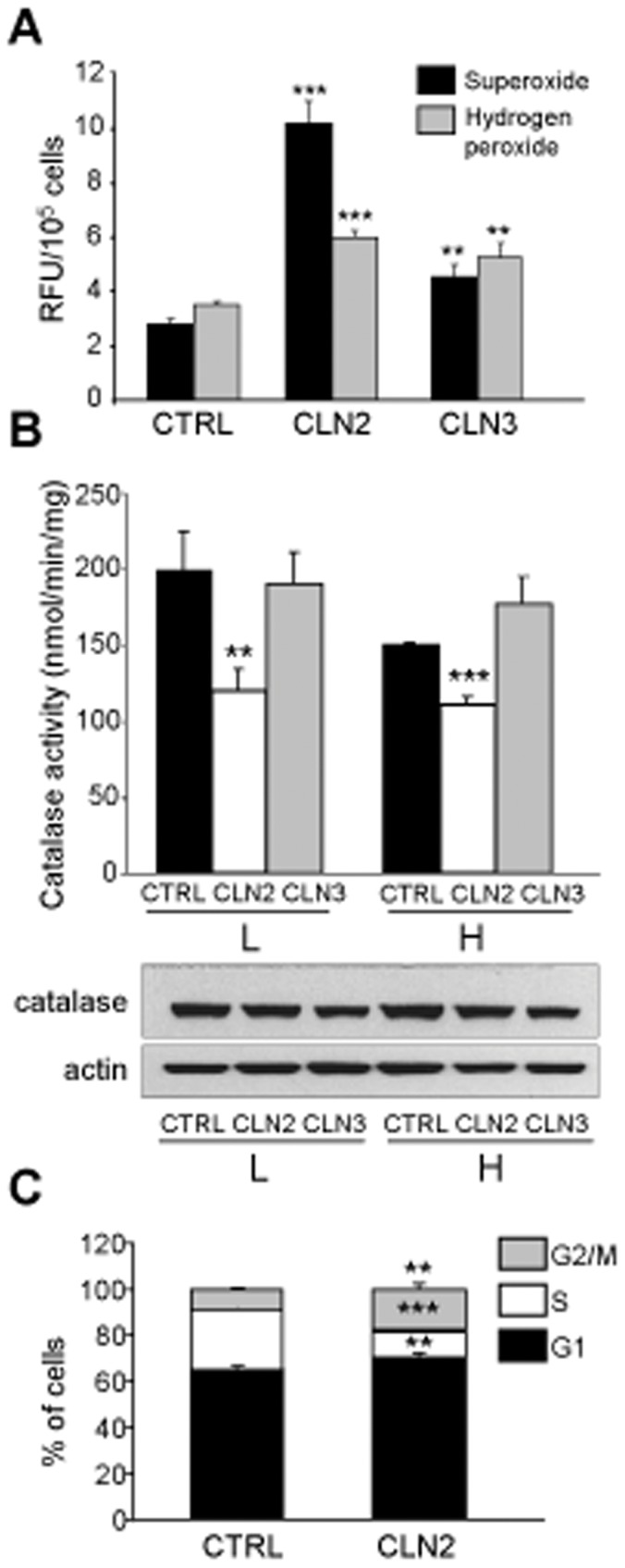
Alterations in oxidative stress in NCL fibroblasts. (A) Superoxide and hydrogen peroxide accumulation. Control, CLN2 and CLN3 fibroblasts incubated under high proteolysis conditions were stained with HE and DHR-123 and analyzed by flow cytometry as described in Materials and Methods. Results are shown as RFU values in 10^5^ cells and represent the mean and S.D. from three separate experiments with triplicated samples. Representative dot blots are shown in Figure S3B. (B) Catalase activities and levels. Control (CTRL), CLN2 and CLN3 fibroblasts were incubated under low (L) and high (H) proteolysis conditions during 3 h. Catalase activity was measured as described in Materials and Methods (upper panel), and lysates (50 µg protein) were immunoblotted with catalase and actin antibodies (lower panels). Catalase activity values represent the mean and S.D. from three separate experiments. (C) Cell cycle analysis. Exponentially growing control (CTRL) and CLN2 fibroblasts, incubated in low proteolysis medium, were stained with PI and the distribution of cells in the different phases of the cell cycle was examined by flow cytometry as described in Materials and Methods. Results are shown as percentage of total cells and represent the mean and S.D. from three separate experiments. Representative dot blots and histograms are shown in Figure S3C. Stars indicate statistically significant differences from control values (**p<0.01 and ***p<0.005).

The observed alterations in macroautophagy can lead to an accumulation of oxidatively damaged mitochondria that in turn fuel the generation of ROS. Mitochondria have been placed at the centre of ROS metabolism and oxidative stress, but peroxisomes are also linked to oxygen metabolism due to the high concentration of hydrogen peroxide generating oxidases and their set of antioxidant enzymes, especially catalase. In order to test if ROS accumulate in CLN2 and CLN3 fibroblasts, cells were stained with HE and with DHR-123, which form fluorescent products with superoxide and hydrogen peroxide respectively, and were then analyzed by flow cytometry as described in Materials and Methods. Superoxide and hydrogen peroxide were markedly and significantly increased in CLN3 and especially in CLN2 fibroblasts in comparison with their controls (Figure 5A and Figure S3B).

To study the origin of ROS, mitochondrial membrane potential was measured, but no significant changes were found (not shown). Next, catalase activity was measured as described in Materials and Methods and it was found to be significantly decreased in CLN2, but not in CLN3 fibroblasts, compared to their controls (Figure 5B). This decrease is not due to lower catalase levels (see western blot in Figure 5B).

To confirm the increased ROS accumulation in CLN2 fibroblasts, we analyzed their cell cycle, because accumulation of ROS usually leads to an arrest of it prior to cell division to avoid the passage of mutated DNA to the following generations. Fibroblasts were stained with PI and subjected to flow cytometry as described in Materials and Methods. Figure 5C and Figure S3C show that CLN2 fibroblasts accumulate in the G2/M phase, about 1.95 times more than in control fibroblasts, at the expense of the S phase, in which the percentage of cells is diminished to about 0.44 times. The percentage of cells in G1 is only slightly (about 1.09 times) increased in CLN2 compared to control fibroblasts. In contrast, there were no significant differences in the cell cycle of CLN3 *vs* control fibroblasts (data not shown). These results are consistent with a larger accumulation of ROS in CLN2 fibroblasts.

### Lysosomal pH is Higher in CLN3 Fibroblasts

**Figure 6:**
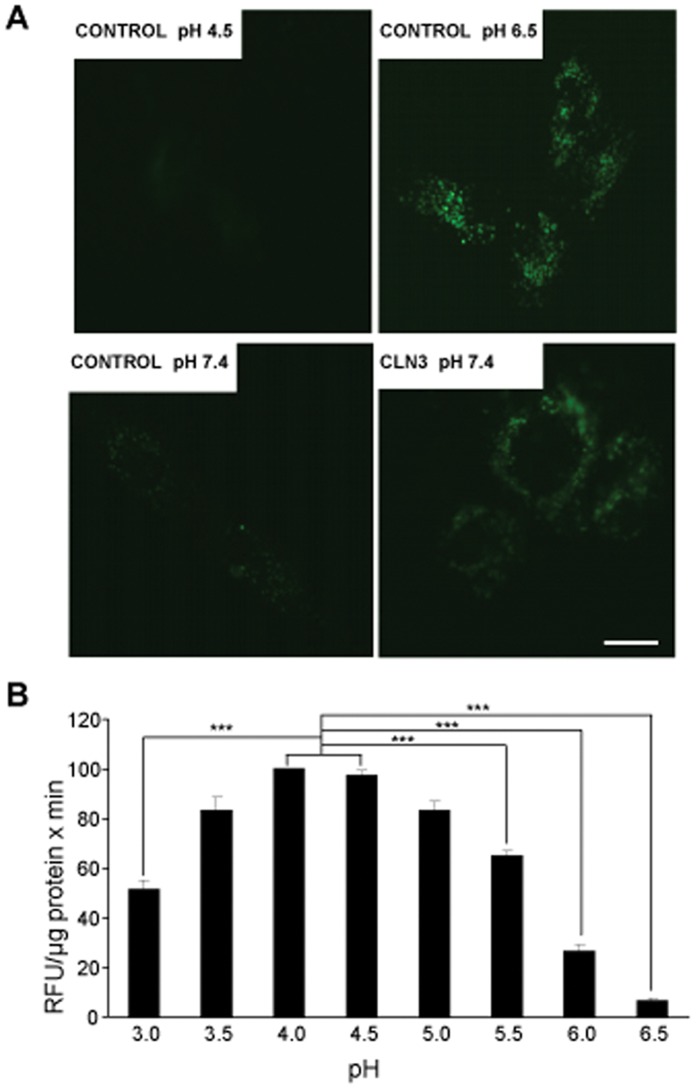
Lysosomes in CLN3 fibroblasts have a higher pH. (A) Exponentially growing control, CLN2 and CLN3 fibroblasts were treated as described in Materials and Methods. Cells were observed by fluorescence microscopy using an inverted microscope and over 200 images were randomly taken at each pH. Upper and lower images show representative photographs of, respectively, permeabilized control fibroblasts incubated in media at pHs 4.5 (left) and 6.5 (right), and non-permeabilized control (left) and CLN3 (right) fibroblasts incubated in high proteolysis medium (pH 7.4). Bar: 20 µm. (B) Effects of different pHs on TPP1 activity. Control fibroblasts were incubated under high proteolysis conditions and TPP1 activity was measured at different pHs using AAF-AMC. Measurements of AMC fluorescence and quantification of protein were carried out as described in Materials and Methods. Results are expressed as RFU values per minute and µg protein and are the mean and S.D. from three separate experiments with triplicated samples. Stars indicate statistically significant differences from pHs 4.0 and 4.5 (***p<0.005).

An increase in lysosomal pH has been shown to reduce the lysosomal degradation capacity and to block autophagosome and endocytic maturation [35]. To test lysosomal pH differences in CLN2 and CLN3 fibroblasts, cells were incubated with FITC-dextran for 24 h, followed by a 1 h chase (Figure 6A). At that time, all fibroblasts were positively marked with FITC-dextran. Quantification of the mean fluorescence intensity of the analyzed cells, as described in the Materials and Methods section, reveals that the lysosomal pH values were 0.5 units higher in CLN3 fibroblasts compared to controls (Table 1). In contrast, no changes were observed in CLN2 fibroblasts. Curiously, lysosomal pHs are greater, in all cases, under high proteolysis conditions (see Table 1), probably reflecting the increased uptake of cytosolic material (which is at neutral pH) that occurs under these conditions. It should be noticed that the increased lysosomal pH observed in CLN3 fibroblasts, compared to CLN2 and control fibroblasts, also affects TPP1 activity, as shown in Figure 6B. This activity is especially impaired (about 60%) at the pH value (6.1±0.2) that occurs in CLN3 lysosomes when the fibroblasts are incubated in a high proteolysis medium. Thus, although TPP1 activity is slightly increased at the optimal pH of the enzyme in CLN3 extracts (see Figure 1B), the activity of this enzyme is strongly reduced in CLN3 fibroblasts due to the increased lysosomal pH of these cells (see Table 1).

**Table 1:** Lysosomal pH values of control, CLN2 and CLN3 fibroblasts incubated in high and low proteolysis media.

	High Proteolysis		Low Proteolysis	
	RFU	pH	RFU	pH
**CONTROL**	78.1±11.7	5.5±0.2	54.1±8.5	4.8±0.2
**CLN2**	67.6±10.3	5.3±0.2	50.6±12.1	4.7±0.3
**CLN3**	117.0±15.2	6.1±0.2**	86.5±10.9	5.5±0.2**

The mean fluorescence intensity values for each cell and condition, obtained in three separate experiments with duplicated samples, were interpolated in the corresponding calibration curves (see Figure S4). Stars indicate statistically significant differences from control values (**p<0.01).

## Discussion

In this study we have attempted to identify similarities and differences in lysosomal functions between the two most frequent forms of NCL, LINCL and JNCL, using patientś fibroblasts (CLN2 and CLN3, respectively). Although macroautophagy disturbances have been already described in JNCL (see e.g. 26), this study demonstrates that an alteration of macroautophagy is common for both NCL phenotypes. This cell defect is worsened by a delay in the arrival of nutrients from the extracellular medium by endocytosis. Two main differences we found between both NCL forms that are relevant for macroautophagy. First, an activation of the Akt-mTOR pathway that affects autophagosome formation in CLN3, but mainly in CLN2 fibroblasts (where p38α and ERK1/2 are also activated). Second, and only in CLN3 fibroblasts, an increase in lysosomal pH that affects autophagosome maturation and, which correlates with the observed increase in LC3-II levels in the absence of lysosomal inhibitors. In addition, the reduced LC3-II levels in CLN2 and CLN3 fibroblasts treated with lysosomal protease inhibitors indicate impairment in autophagosome formation in both cells. These results could be related to the activation of the Akt-mTOR pathway, especially in CLN2 fibroblasts as shown here, by an unidentified signal. The lower activation of p38α, ERK1/2 and Akt in CLN3 compared to CLN2 fibroblasts further suggests that autophagosome maturation is the main autophagic step affected in these cells.

Macroautophagy mediated by mTOR is known to be stimulated by cellular stress, energy depletion, and nutrient deprivation [30], and others have reported energy depletion and reduced ATP levels in the presence of common JNCL mutations [12], [36] and also in a variant of LINCL [14]. Kang *et al.* showed that impairment of macroautophagy in human fibroblasts increased the production of Reactive Oxygen Species (ROS), a known cell stressor, and the accumulation of lipofuscin [37]. In this regard, we report a substantial increase in superoxide and peroxide free radical production, especially in CLN2 fibroblasts in which catalase activity is also reduced. ROS could result from altered mitochondria accumulated in NCL fibroblasts due to defective macroautophagy and could be also responsible for the activation of the Akt-mTOR pathway. Thus, ROS accumulation could be fuelling the inhibition of autophagosome formation in CLN2 and, to a lesser extent, in CLN3 fibroblasts [38].

Although CLN3p has been involved in the regulation of fission and fusion events by membrane protein interactions affecting the traffic of autophagosomes, endosomes, and/or lysosomes [11], [12], its role is still unclear. Our results show that fluid phase endocytosis is more affected in CLN3 than in CLN2 fibroblasts, suggesting an important role of CLN3p in endocytosis. Other studies with yeast and with human fibroblasts have indicated that CLN3p plays an important role in lysosomal pH regulation [7], [39]. We have also found a pH increase of 0.5 units in lysosomes from CLN3, but not from CLN2 fibroblasts. These results could explain the more impaired endocytosis and also the alteration of autophagosomal maturation observed in CLN3 fibroblasts. In fact, LC3-II levels increase in CLN3 fibroblasts in the absence of lysosomal protease inhibitors, indicating a defect in autophagosomal maturation that could result from impaired fusions. In this regard Cao *et al.* [14] also reported a delay in the maturation of autophagosomes to fully degradative autolysosomes in liver cells from homozygous Cln3 ex7/8 mice, showing at the same time that autophagosomes accumulated larger amounts of subunit c of mitochondrial ATP synthase than autolysosomes. Therefore, macroautophagy seems to be at the crossroad of the two different forms of NCL disease, but other considerations should be taken into account, since LINCL patients die earlier than JNCL patients.

In this regard, it is noteworthy that at the pH found in lysosomes from CLN3 fibroblasts (about 0.5 units higher than in lysosomes from control and CLN2 fibroblasts), TPP1 activity is strongly (about 60%) reduced. This compensates the slight increase (about 15%) of TPP1 activity in CLN3 extracts at the optimal pH of the enzyme. TPP1 is known to initiate the degradation, at an optimal lysosomal pH between 4 and 5, of subunit c of mitochondrial ATP synthase, which is also not degraded and accumulates in CLN3 fibroblasts [40]. TPP1 should also degrade other hydrophobic and probably less abundant proteins. It is then possible that as a consequence of the accumulation of hydrophobic proteins, produced by the total or partial defect of TPP1 activity in both NCL forms, lipofuscin accumulates in lysosomes at different paces in LINCL and JNCL and causes a progressive and age-related decline in lysosomal function and macroautophagy. Since lipofuscin accumulation has been shown to decrease the activity of lysosomal hydrolases in human retinal pigment epithelial cells [41], this may be well the initial agent leading to macroautophagy inhibition in NCL diseases.

In spite of the analogies found in the alterations of lysosomal degradation pathways in both forms of NCL, the severity of the symptoms is much worse and the life expectancy is shorter in LINCL than in JNCL patients. Since the most relevant quantitative differences among both CLN2 and CLN3 fibroblasts are in TPP1 activity (fully lost in CLN2 and only partially in CLN3 fibroblasts) and the increased generation of ROS (larger in CLN2 fibroblasts), and both are known to contribute to the accumulation of lipofuscin, these differences could account for the higher severity of LINCL over JNCL. Further analysis is required to establish, besides the differences in TPP1 inhibition and ROS generation, additional triggering events that could lead to the observed alterations in CLN2 and CLN3 fibroblasts and that could explain in more depth the common phenotypes and the differences in onset for both forms of NCL.

## Materials and Methods

### Materials

Minimum essential medium (MEM), human insulin, 3-methyladenine, NH_4_Cl, pepstatin A, ribonuclease A, nigericin, monensin, phenylmethylsulfonyl fluoride (PMSF), EDTA, AAPCMK (Ala-Ala-phenyl-cloromethylketone), Purpald (4-amino-3-hydrazino-5-mercapto-1,2,4-triazole), monodansylcadaverine (Fluka), 40 kDa fluorescein isothiocyanate (FITC)–dextran, bafilomycin A1, Nonidet P40, sodium deoxycholate, paraformaldehyde, MES, Triton X-100 and horseradish-peroxidase labelled secondary antibodies were purchased from Sigma Chemical Co. MEM amino acids 50x, MEM non-essential amino acids 100x, penicillin, MEM vitamins, streptomycin, foetal bovine serum, epidermal growth factor (EGF) and Alexa Fluor 488 goat anti-mouse IgG were supplied by InVitrogen Life Technologies. Leupeptin and lactacystin were acquired from Peptide Institute, Inc. Phospho-specific antibodies that recognize Akt (T-308 or S-463), p38α MAPK (T-180/Y-182), ERK1/2 MAPK (T-202/Y-204), p70S6K (T-389) and 4EBP1 (T-37/46), as well as their pan-specific antibodies, were purchased from Cell Signalling Technologies. Anti-LC3B (clone 5F10) and anti-LAMP1 (clone 14DB) antibodies were purchased from Nanotools and from Abcam, respectively, and anti-catalase and anti-β-actin were from Sigma Chemical Co. Dihydrorhodamine 123 (DHR-123), hydroethidine (HE) and propidium iodide (PI) were purchased from Molecular Probes Inc. [^3^H] valine was obtained from Hartmann Analytic GmbH. A specific substrate of TPP1 enzyme, AAF-AMC (Ala-Ala-phenyl-7-amino-4-methylcoumarin), was purchased from Bachem Bioscience. X-tremeGENE 9 DNA Transfection Reagent, PVDF membranes and Lumi-Light Western Blotting Substrate were from Roche Applied Sciences. FluorSave Reagent was from Calbiochem. Other reagents (hydrogen peroxide, bovine serum albumin, trichloroacetic acid, chloroacetate, etc.) were of the best analytical quality available. The pEGFP-LC3 expression vector was a gift from Dr. N. Mizushima (The Tokyo Metropolitan Institute of Medical Sciences, Tokyo, Japan).

### Cell Culture

In this study we used the following fibroblasts from the Coriell Institute for Medical Research (Camden, NJ, USA): GM03349 (a 10 years old control male), GM0048 (a 10 years old control male), GM01582 (a 12 years old control female), GM09404 (a female CLN2 patient with an I287N mutation) and GM16486 (a male CLN2 patient, one allele with an R127Q missense mutation in exon 4 and a second allele with an IVS5-1G>C splice defect). In addition, fibroblasts were also obtained from two Spanish patients: MPA (a 5 years old male CLN2 patient with an S475L mutation) and MPL (a 13 years old male CLN3 patient with the 1.02 kb deletion that is the most prevalent mutation in JNCL). Cells were grown at 37°C in a humidified atmosphere of 5% (v/v) CO_2_/air in MEM with Earle’s salts, 2 mM L-glutamine, 1x MEM amino acids, 1x MEM non-essential amino acids, 1x MEM vitamins, 100 U/ml penicillin, 100 µg/ml streptomycin with 15% foetal bovine serum (fibroblasts’ growth medium). Cell viability and growth curves were obtained by counting the cells with a hemocytometer chamber and using the trypan blue exclusion test. All experiments were performed at passage number 10–14 to avoid culture aging effects. Krebs–Henseleit medium (KH: 118.4 mM NaCl, 4.75 mM KCl, 1.19 mM KH_2_PO_4_, 2.54 mM MgSO_4_, 2.44 mM CaCl_2_•2H_2_O, 28.6 mM NaHCO_3_ and 10 mM glucose with 10 mM Hepes, pH 7.4), was used for high proteolysis (starvation) conditions. For low proteolysis conditions, fibroblasts’ growth medium or KH containing insulin 0.1 mM and essential amino acids at two times the concentration present in the fibroblastś growth medium were used [32], [33].

### Measurements of Intracellular Protein Degradation

Experiments were carried out as described [32], [33]. Briefly, short- or long-lived proteins were labelled for 15 min or 24 h with 5 or 1 µCi/ml [^3^H] valine, respectively. Then, fibroblasts were washed three times with fibroblasts’ growth medium containing 10 mM L-valine. The net release of trichloroacetic acid-soluble radioactivity from the labelled cells into the culture medium was analyzed for the indicated times and culture conditions, starting either immediately (short-lived proteins) or after a 24 h chase in fibroblasts’ growth medium containing 10 mM L-valine (long-lived proteins) to degrade the short-lived proteins. Protein degradation was expressed as percentage of the initial protein that is degraded per hour, and proteolytic rates were calculated by least-squares regression of semi-logarithmic plots. The contribution of the various proteolytic pathways was calculated using 20 mM NH_4_Cl and 0.1 mM leupeptin (for total lysosomes: macroautophagy, microautophagy, chaperone-mediated autophagy, etc., see [42]), 10 mM 3-methyladenine (for macroautophagy) and 10 µM lactacystin (for proteasomes), as previously described [32].

### Measurements of Autophagic Flux

To assess autophagy by LC3-II levels, confluent fibroblasts were incubated under high and low proteolysis conditions (see above, Cell culture), with or without lysosomal inhibitors. Either bafilomycin A_1_ (500 nM) or ClNH_4_ (20 mM) plus leupeptin (0.1 mM) were used [43] for the last 2 h with similar results.

Cells were homogenized in RIPA buffer (150 mM NaCl, 1% Nonidet P40, 0.5% sodium deoxycholate, 0.1% SDS, 50 mM Tris, pH 8.0) containing 0.1 mM leupeptin and 1 mM PMSF. Crude extracts were centrifuged at 12,000 g for 10 min at 4°C and the supernatants were collected for western blot experiments. Proteins (75 µg) from the various lysates were separated on 15% SDS-PAGE gels, transferred to PVDF membranes and analyzed by immunoblot with anti-LC3B antibodies. The housekeeping protein actin was used to normalize the results.

To assess autophagy by pEGFP-LC3 fluorescence, cells were plated in 24-well dishes at a density of 80,000 cells per well for 24 h. Then, they were transfected with 1.5 µg pEGFP-LC3, using the X-tremeGENE 9 DNA Transfection Reagent according to the manufacturer’s instructions. The transfection mixture was incubated 8 h at 37°C and replaced by fibroblasts’ growth medium for 48 h. Cells were fixed for 10 min with phosphate buffered saline (PBS) containing 4% paraformaldehyde and mounted on microscope slides using FluorSave Reagent and coverslips.

For analysing the cells with EGFP-LC3 vesicles, the number of EGFP-LC3 dots in thirty EGFP-positive cells per cell line was counted. We considered a cell EGFP-positive if it had more than five LC3 fluorescent vesicles [44].

For the monodansylcadaverine experiments cells were incubated for 3 h at 37°C in high proteolysis medium. In the last 15 min, monodansylcadaverine was added at 100 µM (final concentration) and, after three washes with ice-cold PBS, the cells were mounted on microscope slides as above. Monodansylcadaverine was measured (excitation wavelength 380 nm, emission wavelength 525 nm) in an M5/SpectraMax microplate reader from Molecular Devices and expressed as % specific activity (arbitrary units) as described [45]. Fluorescence (EGFP and monodansylcadaverine) was observed (40x oil immersion lens) using a fluorescence microscope Observer Z.1 coupled with an ApoTome optical sectioning system (Carl Zeiss, Inc.).

### Immunocytochemistry of LAMP1

For immunocytochemistry of LAMP1, cells on glass coverslips were fixed for 10 min with PBS containing 4% paraformaldehyde. After PBS washing, the cells were permeabilized with 0.1% Triton X-100/PBS for 20 min at room temperature, washed in PBS and blocked with 4% foetal bovine serum/PBS for 30 min. Cells were incubated overnight at 4°C with anti-LAMP1 antibody, and a secondary antibody conjugated to Alexa 488 was applied for 1 h at room temperature, both to a final concentration 1∶500. The slides were mounted and observed as above.

### TPP1 Activity Measurements

Exponentially growing cells were homogenized with 50 mM sodium acetate buffer, pH 4.0, and 0.1% Triton X-100 plus protease inhibitors (1 mM PMSF, 100 µM leupeptin, 1 µM pepstatin A and 5 mM EDTA). Lysates were centrifuged at 12,000 g for 25 min, 4°C. Protein in supernatants was measured by a Lowry-deoxycholate method [46], using 10 µg protein per assay. AAF-AMC (100 µM) was used as TPP1 substrate and the reaction was carried out for 30 min in 0.1 M sodium acetate buffer, pH 4.0, with 0.1% Triton X-100 in a final volume of 100 µl and stopped by adding 50 µl of 100 mM sodium chloroacetate and 20 mM sodium acetate. TPP1 activity was calculated by subtracting the fluorescence obtained in the presence of 1 µM AAPCMK, a specific TPP1 inhibitor, from the fluorescence obtained in its absence. Fluorescences were measured in an M5/SpectraMax microplate reader from Molecular Devices and the results were normalized to the protein content per assay. For measurements of pH effect on TPP1 enzymatic activity, sodium acetate or MES buffers were prepared at several pH values, ranging from 3 to 6.5. Changing the buffer for a given pH did not affect the enzymatic activity.

### Lysosomal pH Measurements

Fibroblasts were grown in high or low proteolysis media that had been previously adjusted to several pHs, ranging from 4.5 to 7.0, with sodium acetate or MES buffers. They were incubated in their respective high or low proteolysis media containing 0.5 mg/ml FITC-dextran for 24 h, washed twice with PBS and chased for 1 h with the corresponding fresh medium without FITC-dextran. To allow the lysosomal pH to match the medium pH, before analysis the cells were previously treated with 10 µM nigericin and 20 µM monensin for 1 h. At each pH value, 200 cells were randomly chosen, photographs were taken with an Observer Z.1 fluorescence microscope coupled with an ApoTome optical sectioning system and the mean fluorescence intensity was quantified using the Carl Zeiss Software (Axio Vision 4.6). Fluorescence (excitation: 495 nm, emission: 519 nm) expressed in Relative Fluorescence Units (RFU) was determined for each individual cell, subtracting the corresponding background. RFU values *vs* pH were linearized and plotted in each calibration curve (Figure S4) for high and low proteolysis experimental conditions. Lysosomal pH of human fibroblasts was calculated by interpolating the mean fluorescence intensity per condition in its respective calibration curve.

### Oxidative Stress Assays and Cell Cycle Measurements

For quantification of mitochondrial ROS, cells were incubated for 1 h at 37°C in high proteolysis medium. Then, 5 µM HE or 5 µM DHR-123 were added and the cells were further incubated for 30 min at 37°C, washed twice with PBS and trypsinized. About 5×10^5^ cells were collected per sample in high proteolysis medium, which were immediately analyzed by flow citometry (FACSCalibur flow cytometer from BD Biosciences). Mean fluorescence values were obtained at an excitation and emission wavelengths of 505 and 595 nm (for HE) and 485 and 525 nm (for DHR-123), respectively. Catalase activity was measured in total extracts from cells incubated under low and high proteolysis conditions in a microplate reader M5/SpectraMax, as previously described [47]. Enzyme levels were semiquantitatively determined by western blot.

For cell cycle determinations, about 2×10^6^ cells were pelleted at 16,000 g for 1 min and were fixed overnight at 4°C with ice-cold 70% ethanol. The cell pellet was washed twice in 500 µl of 20 mM EDTA, pH 8.0, incubated 10 min with 0.1 mg/ml ribonuclease A in 500 µl of 20 mM EDTA, pH 8.0, and the cells were stained with PI (40 µg/ml) for 30 min at 37°C. Stained cells were analyzed by flow cytometry in a FACSCalibur flow cytometer from BD Biosciences at excitation and emission wavelengths of 535 and 617 nm, respectively. The DNA histograms were analyzed by the FlowJo Software (Tree Star Inc, Ashland, Oregon, U.S.A.) and the percentages of cells with different DNA content were determined.

### General Procedures

Protein concentrations were determined using a BCA Protein Assay Kit (Pierce) according to the manufacturer’s instructions or a Lowry-deoxycholate method [46]. For analysis of protein phosphorylation by immunoblot, cells were collected and lysed in mammalian lysis buffer (150 mM NaCl, 1% Nonidet-P40, 50 mM Tris-HCl, 0.5% sodium deoxycholate, 0.1% SDS, 15% glycerol, 1 mM PMSF, 100 µM leupeptin, 2 mM Na_3_VO_4_, 100 mM NaF and 20 mM Na_4_P_2_O_7_, pH 7.4) at 4°C. Phospho-specific antibodies were always used in the first round of immunoblotting. After treating the membranes overnight at 4°C with stripping buffer (0.1 M glycine, pH 2.3), they were probed using the pan-specific antibodies. Blots were then developed using Lumi-Light Western Blotting Substrate. Protein bands were quantified by densitometric analysis with an Image Quant ECL (GE Healthcare). Electron microscopy was carried out as detailed in [48]. The determination by flow cytometry of fluid phase endocytosis time course was performed essentially as described earlier [49]. Briefly, cells were incubated for the indicated times at 37°C with FITC-dextran (0.5 mg/ml) in fibroblasts’ growth medium. Then, cells were washed with cold PBS, collected at 4°C and about 5×10^5^ cells per sample were resuspended in cold PBS. The obtained cells were immediately analyzed by flow cytometry in a FACSCalibur flow cytometer from BD Biosciences. Cells were analyzed at excitation and emission wavelengths of 495 and 519 nm and the % of FITC-dextran positive cells was calculated for every experimental condition. Data for the different conditions are presented as means ± S.D. of at least three independent experiments. Data for controls and CLN2 fibroblasts are pooled from the three different cell lines in each case, since we didńt noticed significant differences between them. The data for CLN3 fibroblasts are from a single patient (see Cell culture). Statistical analysis was performed using Graph Pad Prism version 5.0 statistical software. Differences between paired samples were analyzed by two-tailed Student’s t-tests and, for comparisons of more samples, one-way ANOVA was used, followed by Student-Newman-Keuls post hoc test. The significance has been considered at *p<0.05, *p<0.01 and ***p<0.005, as indicated in each case.

## Supporting Information

Figure S1 **Protein degradation in NCL fibroblasts.** Long-lived (upper panel) and short-lived (lower panel) proteins were radioactively labelled in control, CLN2 and CLN3 fibroblasts, and protein degradation was analyzed under the indicated conditions as described in Materials and Methods. Results are presented as the percentage of the labelled protein that is degraded per hour and are the mean and S.D. from twelve to fifteen separate experiments with duplicated samples. No significant differences between cell lines were found.
(TIF)

Figure S2 **Monodansylcadaverine staining of lysosomes.** Control, CLN2 and CLN3 fibroblasts were incubated under high and low proteolysis conditions with 100 µM monodansylcadaverine for 15 min at 37°C. (A) Representative fluorescent images obtained as described in Materials and Methods from the fibroblasts incubated under high proteolysis conditions. Bar: 20 µm. (B) Monodansylcadaverine was measured under high and low proteolysis conditions as described in Materials and Methods. Results are shown as percentage of monodansylcadaverine specific activity (arbitrary units) and represent the mean and S.D. from three separate experiments. Stars immediately on top of bars indicate statistically significant differences from control values (*p<0.05, **p<0.01 and ***p<0.005).
(TIF)

Figure S3 **Dot blots and histograms from the various cytometric analyses.** (A) FITC-dextran internalization (see Figure 3B). Representative dot blots showing the entry of FITC-dextran in control (CTRL), CLN2 and CLN3 fibroblasts. In the y axes, forward scatter values are represented in a linear scale and in the x axes the fluorescence intensity values in the FL 1 channel corresponding to the FITC-dextran fluorescence are represented in a logarithmic scale. (B) ROS determination (see Figure 5A)**.** Representative dot blots of HE and DHR-123 staining in control (CTRL), CLN2 and CLN3 fibroblasts are shown, plotting in the y axes the forward scatter values in a linear scale and in the x axes the fluorescence intensity values in the FL 1 channel (DHR123) and in the FL 3 channel (HE) in a logarithmic scale. (C) Cell cycle analysis (see Figure 5C). Control (CTRL) and CLN2 fibroblasts were stained with PI and the distribution of the cells in the different phases of the cell cycle was examined. Representative dot blots and histograms are shown. In the dot blots, the pulse widths of the PI fluorescence signals are represented in the y axes and the fluorescence intensities of the FL 3 channel (PI fluorescence) in the x axes. In the histograms, the numbers of analyzed cells are represented in the y axes and the PI fluorescence intensity in the x axes. The different phases of the cell cycle are depicted as B (G1), C (S) and D (G2/M). E depicts the cells that underwent apoptosis (subG0/G1).
(TIF)

Figure S4 **Calibration curves of intralysosomal pH.** Calibration curves for FITC-dextran under pHs from 4.5 to 7.0 were obtained in high and low proteolysis media as described in Materials and Methods. Regression equations and the correlation coefficients are shown.
(TIF)
