# Supplementary figures and images for: Alterations in ROS Activity and Lysosomal pH Account for Distinct Patterns of Macroautophagy in LINCL and JNCL Fibroblasts
Source: PLoS One. 2013 Feb 7;8(2):e55526. doi: 10.1371/journal.pone.0055526 (PMC3567113; doi:10.1371/journal.pone.0055526)

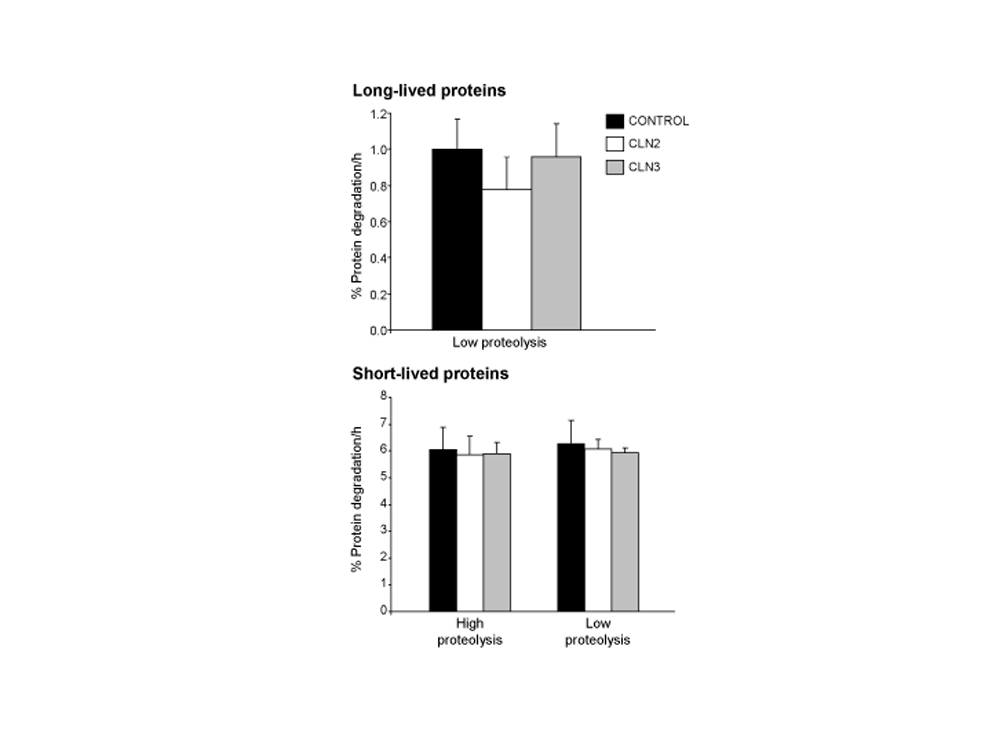

Supplement: Figure S1 — Protein degradation in NCL fibroblasts. Long-lived (upper panel) and short-lived (lower panel) proteins were radioactively labelled in control, CLN2 and CLN3 fibroblasts, and protein degradation was analyzed under the indicated conditions as described in Materials and Methods. Results are presented as the percentage of the labelled protein that is degraded per hour and are the mean and S.D. from twelve to fifteen separate experiments with duplicated samples. No significant differences between cell lines were found. (TIF) [file pone.0055526.s001.tif]

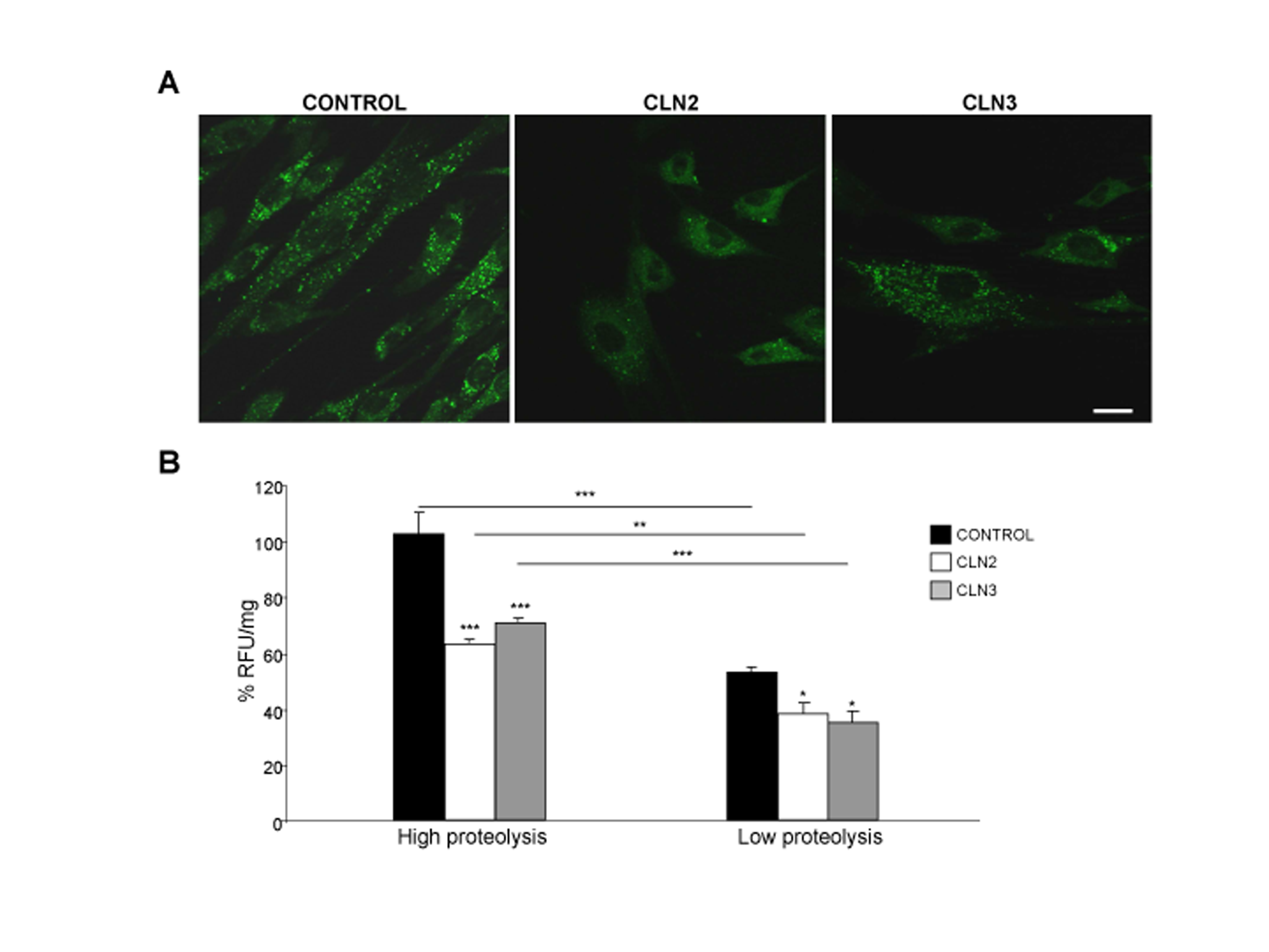

Supplement: Figure S2 — Monodansylcadaverine staining of lysosomes. Control, CLN2 and CLN3 fibroblasts were incubated under high and low proteolysis conditions with 100 µM monodansylcadaverine for 15 min at 37°C. (A) Representative fluorescent images obtained as described in Materials and Methods from the fibroblasts incubated under high proteolysis conditions. Bar: 20 µm. (B) Monodansylcadaverine was measured under high and low proteolysis conditions as described in Materials and Methods. Results are shown as percentage of monodansylcadaverine specific activity (arbitrary units) and represent the mean and S.D. from three separate experiments. Stars immediately on top of bars indicate statistically significant differences from control values (*p<0.05, **p<0.01 and ***p<0.005). (TIF) [file pone.0055526.s002.tif]

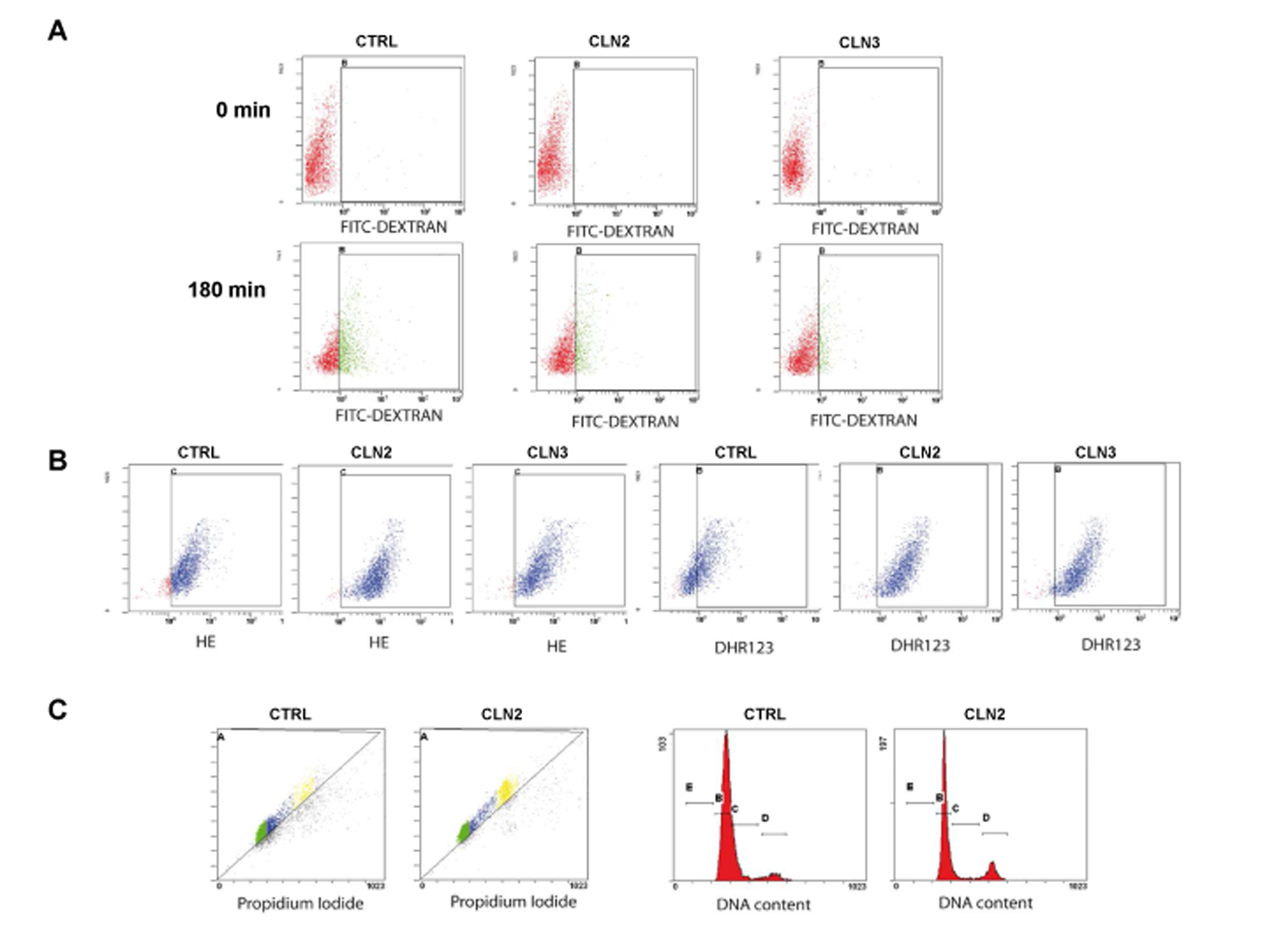

Supplement: Figure S3 — Dot blots and histograms from the various cytometric analyses. (A) FITC-dextran internalization (see Figure 3B). Representative dot blots showing the entry of FITC-dextran in control (CTRL), CLN2 and CLN3 fibroblasts. In the y axes, forward scatter values are represented in a linear scale and in the x axes the fluorescence intensity values in the FL 1 channel corresponding to the FITC-dextran fluorescence are represented in a logarithmic scale. (B) ROS determination (see Figure 5A). Representative dot blots of HE and DHR-123 staining in control (CTRL), CLN2 and CLN3 fibroblasts are shown, plotting in the y axes the forward scatter values in a linear scale and in the x axes the fluorescence intensity values in the FL 1 channel (DHR123) and in the FL 3 channel (HE) in a logarithmic scale. (C) Cell cycle analysis (see Figure 5C). Control (CTRL) and CLN2 fibroblasts were stained with PI and the distribution of the cells in the different phases of the cell cycle was examined. Representative dot blots and histograms are shown. In the dot blots, the pulse widths of the PI fluorescence signals are represented in the y axes and the fluorescence intensities of the FL 3 channel (PI fluorescence) in the x axes. In the histograms, the numbers of analyzed cells are represented in the y axes and the PI fluorescence intensity in the x axes. The different phases of the cell cycle are depicted as B (G1), C (S) and D (G2/M). E depicts the cells that underwent apoptosis (subG0/G1). (TIF) [file pone.0055526.s003.tif]

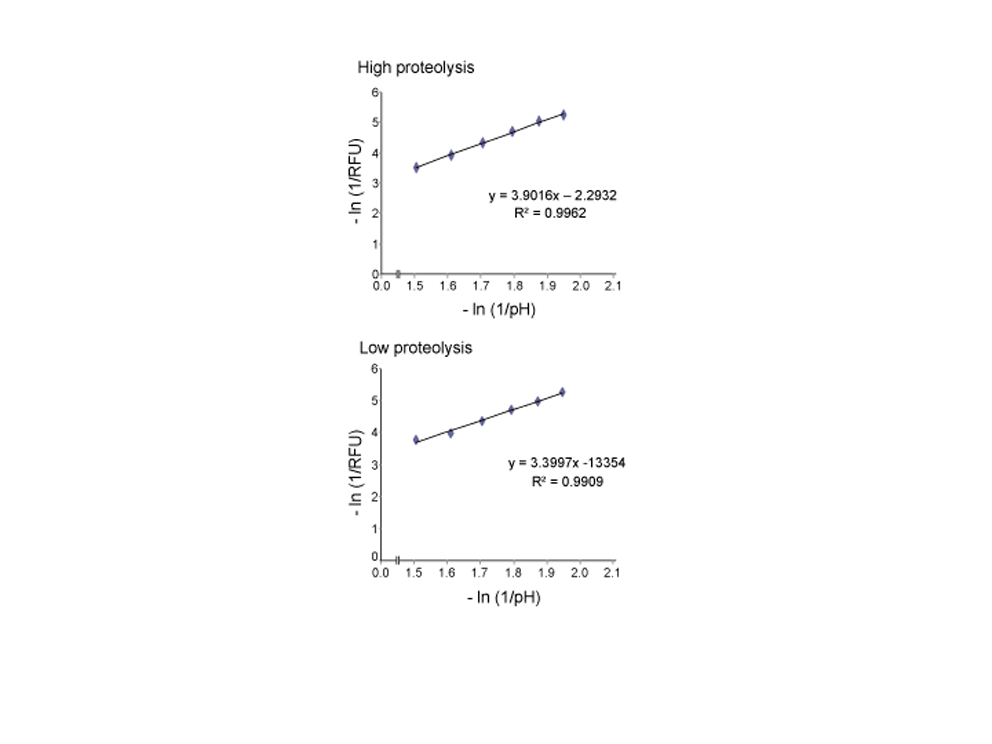

Supplement: Figure S4 — Calibration curves of intralysosomal pH. Calibration curves for FITC-dextran under pHs from 4.5 to 7.0 were obtained in high and low proteolysis media as described in Materials and Methods. Regression equations and the correlation coefficients are shown. (TIF) [file pone.0055526.s004.tif]
